# The flow cytometry-defined light chain cytoplasmic immunoglobulin index and an associated 12-gene expression signature are independent prognostic factors in multiple myeloma

**DOI:** 10.1038/leu.2015.65

**Published:** 2015-03-27

**Authors:** X Papanikolaou, D Alapat, A Rosenthal, C Stein, J Epstein, R Owens, S Yaccoby, S Johnson, C Bailey, C Heuck, E Tian, A Joiner, F van Rhee, R Khan, M Zangari, Y Jethava, S Waheed, F Davies, G Morgan, B Barlogie

**Affiliations:** 1 Myeloma Institute for Research and Therapy, University of Arkansas for Medical Sciences, Little Rock, AR, USA; 2 Department of Pathology, University of Arkansas for Medical Sciences, Little Rock, AR, USA; 3 Cancer Research and Biostatistics, Seattle, WA, USA

## Abstract

As part of Total Therapy (TT) 3b, baseline marrow aspirates were subjected to two-color flow cytometry of nuclear DNA content and cytoplasmic immunoglobulin (DNA/CIG) as well as plasma cell gene expression profiling (GEP). DNA/CIG-derived parameters, GEP and standard clinical variables were examined for their effects on overall survival (OS) and progression-free survival (PFS). Among DNA/CIG parameters, the percentage of the light chain-restricted (LCR) cells and their cytoplasmic immunoglobulin index (CIg) were linked to poor outcome. In the absence of GEP data, low CIg <2.8, albumin <3.5 g/dl and age ⩾65 years were significantly associated with inferior OS and PFS. When GEP information was included, low CIg survived the model along with GEP70-defined high risk and low albumin. Low CIg was linked to beta-2-microglobulin >5.5 mg/l, a percentage of LCR cells exceeding 50%, C-reactive protein ⩾8 mg/l and GEP-derived high centrosome index. Further analysis revealed an association of low CIg with 12 gene probes implicated in cell cycle regulation, differentiation and drug transportation from which a risk score was developed in TT3b that held prognostic significance also in TT3a, TT2 and HOVON trials, thus validating its general applicability. Low CIg is a powerful new prognostic variable and has identified potentially drug-able targets.

## Introduction

DNA flow cytometry detects aneuploidy in 70–80% of patients with multiple myeloma (MM).^1^ Hypo-diploidy has been associated with poor prognosis in patients treated with VAD (vincristine, doxorubicin and dexamethasone)^2^ that was overcome by the use of high-dose melphalan.^3^ In contrast, hyperdiploidy has been associated with more favorable outcomes.^4, 5^ Here we have investigated, as part of Total Therapy 3b,^6^ the prognostic implications of two-color flow cytometry of nuclear DNA and cytoplasmic immunoglobulin (DNA/CIG) parameters in the context of all standard prognostic variables and plasma cell-based gene expression profiling (GEP).

## Patients and methods

### Treatment, staging and clinical endpoints

The details of the TT3b trial and clinical outcomes have been reported previously.^6^ Briefly, 177 eligible patients with newly diagnosed MM fulfilling CRAB criteria^7^ were enrolled, including 26 with one cycle of prior therapy. The protocol consisted of two induction cycles with VTD-PACE (bortezomib, thalidomide, dexamethasone and 4-day continuous infusions of cisplatin, doxorubicin, cyclophosphamide and etoposide) with hematopoietic progenitor cell collection upon recovery from the first cycle. Melphalan 200 mg/m^2^ was applied with each of the planned two transplants, with dose adjustments for age and renal function.^8^ Consolidation employed dose-reduced VTD-PACE for two cycles. Maintenance with VRD (bortezomib, lenalidomide and dexamethasone) was planned for 3 years. In compliance with the institutional, federal and Helsinki declaration guidelines, all patients provided written informed consent before enrollment into the protocol that had been approved by the institutional review board.

All patients underwent a standardized staging workup. Bone marrow examinations included DNA/CIG, metaphase karyotyping to document the presence of cytogenetic abnormalities and GEP of purified plasma cells to assign molecular subclass,^9^ risk according to 70 (ref. 10) and 80 gene models,^11^ GEP-defined 1q21 amplification (amp1q21) as well as proliferation index^9^ and centrosome index.^12^ Clinical endpoints included the frequency of complete response^13^ and its duration counted from complete response onset to progression or death from any cause. Overall survival (OS) and progression-free survival (PFS) were measured from start of protocol therapy until progression or death from any cause for PFS and death from any cause for OS. Outcome data were updated as of 21 February 2014.

### DNA/CIG assay

As part of the diagnostic workup, DNA/CIG was performed in all Total Therapy (TT) protocols with continuous updates on hardware and methodology. A modification introduced in August 2006 on the doublet discrimination method^14^ increased accuracy and reproducibility of results and has been uniformly applied with the start of TT3b enrollment. Details of the DNA/CIG method have been published.^1^ Briefly, bone marrow aspirates were separated by Hypaque-Ficoll (Sigma Aldrich, St Louis, MO, USA) gradient centrifugation, erythrocytes lysed with ammonium chloride and samples submitted to overnight ethanol fixation. Single-cell suspensions were exposed to anti-light chain reagents (Dako Kappa and Lambda light chain (Agilent Technologies/Dako, Glostrup, Denmark) F(AB)_2_/FITC conjugated) and then counterstained for DNA with propidium iodide with the addition of RNase. Acquisition and analysis of the flow cytometric signals for the derived parameters were done through a BD FACScan Flow Cytometer (Beckton, Dickinson and Company, Franklin Lakes, NJ, USA) and the CellQuest/CellFit software (Beckton, Dickinson and Company). Routinely, a total of at least 10 000 events were recorded and analyzed. Assays with fewer than 500 events were rejected. To ensure maximum reproducibility of results, the same instrument was used for all measurements. The instrument was standardized daily with DNA Check Beads (Beckman Coulter, Inc., Brea, CA, USA) for consistent channel settings and coefficient of variation requirements of <3%. A known positive patient specimen for each light chain was run daily and percent positive and light chain intensity were recorded. Titrated polyclonal F(AB')_2_ antibodies for light chains were used for low nonspecific binding, and excellent lot-to-lot reproducibility was documented. To quantitate the cellular DNA content, the DNA index (DI)^15^ was determined and calculated as the ratio of the mean for each light chain-positive G0/1 DNA peak divided by the mean of the light chain-negative diploid G0/1 peak on the *x*-axis.

Acquisition of the G0/G1 populations was done through the modified doublet discrimination method^14^ and the CellQuest/CellFit software. A DI between 0.99 and 1.01 was referred to as diploid, whereas hyperdiploid implied DI >1.01 and hypodiploid DI <0.99. The excess of kappa- or lambda-positive cells identified the involved or light chain-restricted (LCR) cell population, the percentage of which was calculated in relation to the total number of gated events. Among the LCR cell population, discrete populations of cells with different nuclear DNA content were identified, which we refer to from here on as DNA stem lines, and their respective percentage could be calculated by referral to the total number of gated events. The involved DNA stem line with the highest percentage was considered dominant. The ploidy status was characterized from the DI of the dominant LCR DNA stem line. To quantitate the cytoplasmic immunoglobulin content of a light chain-positive population, the cytoplasmic immunoglobulin index (CIg) was used and calculated from the ratio of the geometric mean of the *y*-axis (cytoplasmic immunoglobulin fluorescence intensity) for the light chain-positive G0/1 peak divided by the *y*-axis geometric mean of the light chain-negative diploid G0/1 population. The CIg of each distinct DNA stem line was calculated as explained above. An example of a kappa-positive hyperdiploid MM with two distinct stem lines along with a case of high and a case of low CIg are shown in Supplementary Figures 1 and 2.

There was absolute concordance between the light chain classification of the LCR population by FDC and the conventional serological methods. In addition, the dominant CIg correlated with the ratio of M-protein to the percentage the dominant stem line (*R*_S_=0.621, *P*<0.001). Although the DNA/CIG method described here does not discriminate between mature B cells and plasma cells, it does include all the myeloma cell subpopulations that have either an aberrant phenotype^16^ or a dim expression of the selected antigen^17, 18^ or that even belong to the rare category of nonsecreting and nonproducing myeloma cells.^1^ When multiparameter flow cytometry was performed to identify LCR non-plasma B cells, their percentage was consistently found to be <1%.^19^

### Statistical Analysis

Kaplan–Meier methods were used to generate survival distribution graphs, and comparisons were made employing the log-rank test. The Pearson *χ*^2^-test was used for categorical comparisons, whereas Student's *t*-test and Mann–Whitney *U*-test were used to compare the means or medians, respectively, of two different populations. Spearman's rank correlation coefficient (*R*_S_) was used as a measure of association between the ranks of two variables. For continuous variables, the running log-rank method was applied for the calculation of optimal cutoff points.^20^ Stepwise selection and Cox proportional hazard regression modeling were applied in multivariate analyses. The R^2^ statistic was used to evaluate the predictive power of different models.^21^ For the identification of differentially expressed gene probe sets between dichotomized groups, the Wilcoxon's rank sum test of significance analysis of microarrays^22^ was used with an adjustment of a false discovery rate (or *q*-value) of <10% to be considered significant. The logarithmic base 2 expression levels of the gene probe sets were used in the analyses. Microarray data used in this study have been deposited in the NIH Gene Expression Omnibus under accession number GSE2658. A modified approach to the ComBat method^23^ was used to transform HOVON gene expression data to the same scale as TT3b while keeping the TT3b gene expression data fixed.

## Results

Standard baseline characteristics were available in 173 of 177 patients enrolled; in addition, 166 had GEP and 143 had DNA/CIG data. Herein we report on the 139 patients with complete data sets for both DNA/CIG and GEP analyses (Table 1). Standard variables and GEP data did not differ from the larger patient sets (data not shown) but, compared with earlier TT trials, cytogenetic abnormalities (42%) and GEP-70-defined high risk (23%) were more frequent. Aneuploidy was detected in 88%. DNA stem line frequencies were 1 in 18%, 2 in 70% and >2 in 12%. In case of multiple LCR DNA stem lines, the designations of hyperdiploid applied to 58%, diploid to 38% and hypodiploid to 4%. There was absolute concordance between the light chain classification of the LCR population by FDC and the conventional serological methods. Moreover, there was a substantial correlation (R_S_=0.621, *P*<0.001) between the dominant CIg and the ratio of M-protein to the percentage of that stem line.

**Figure 1:**
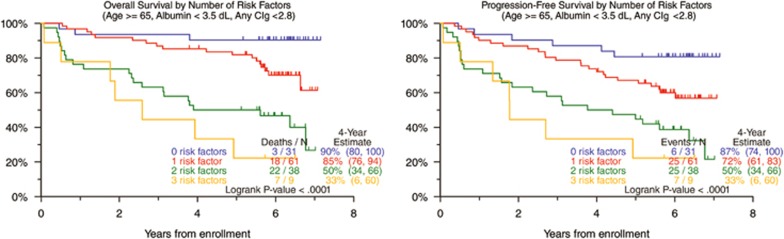
Kaplan–Meier plots of OS and PFS in TT3b as defined by the multivariate survival analysis model (GEP variables excluded).

Clinical outcomes for the 139 patients of the TT3b study are shown in Supplementary Figure 3. In a univariate analysis, 4-year estimates were 73% for OS, 67% for PFS and 69% for complete response duration among the 67% achieving complete response . Both OS and PFS were inferior with low levels of albumin <3.5 g/dl and high levels of beta-2-microglobulin >5.5 mg/l and lactate dehydrogenase ⩾190 U/l (Table 2). Both GEP70 and GEP80 high-risk designations were associated with poor OS and PFS. Other adverse GEP variables included PR subgroup, proliferation index ⩾10, centrosome index ⩾3 and amp1q21. Among DNA/CIG-derived parameters, adverse prognostic implications were linked to cases with >2 DNA stem lines, LCR ⩾50% and low CIg <2.8 (optimal cutoff point derived from running log-rank analysis on PFS), regardless of DNA stem line dominance. Next, we performed several multivariate analyses. In the absence of GEP data (model 1), low albumin, older age and low CIg were associated with shorter OS and PFS. The combined effect of the presence of these variables is depicted in Figure 1. When GEP variables were also considered (model 2), low albumin, low CIg and age maintained their independent prognostic significance. New variables entering the model included GEP70-defined high risk, proliferation index, and—for PFS only—IgA isotype.

Given the association of CIg with poor survival in this trial, we examined the variables linked to low CIg (<2.8; Table 3). With the exception of low albumin, low CIg was linked to all adverse standard parameters (beta-2-microglobulin, C-reactive protein, lactate dehydrogenase, hemoglobin, marrow plasmacytosis and cytogenetic abnormalities). Significant associations were also noted between low CIg and GEP-defined high risk (both GEP70 and GEP80), centrosome index and LCR%. The MS molecular subgroup was under-represented in patients with low CIg. High beta-2-microglobulin and C-reactive protein, centrosome index ⩾3 and LCR exceeding 50% were independently and positively linked to low CIg in multivariate analysis.

**Figure 2:**
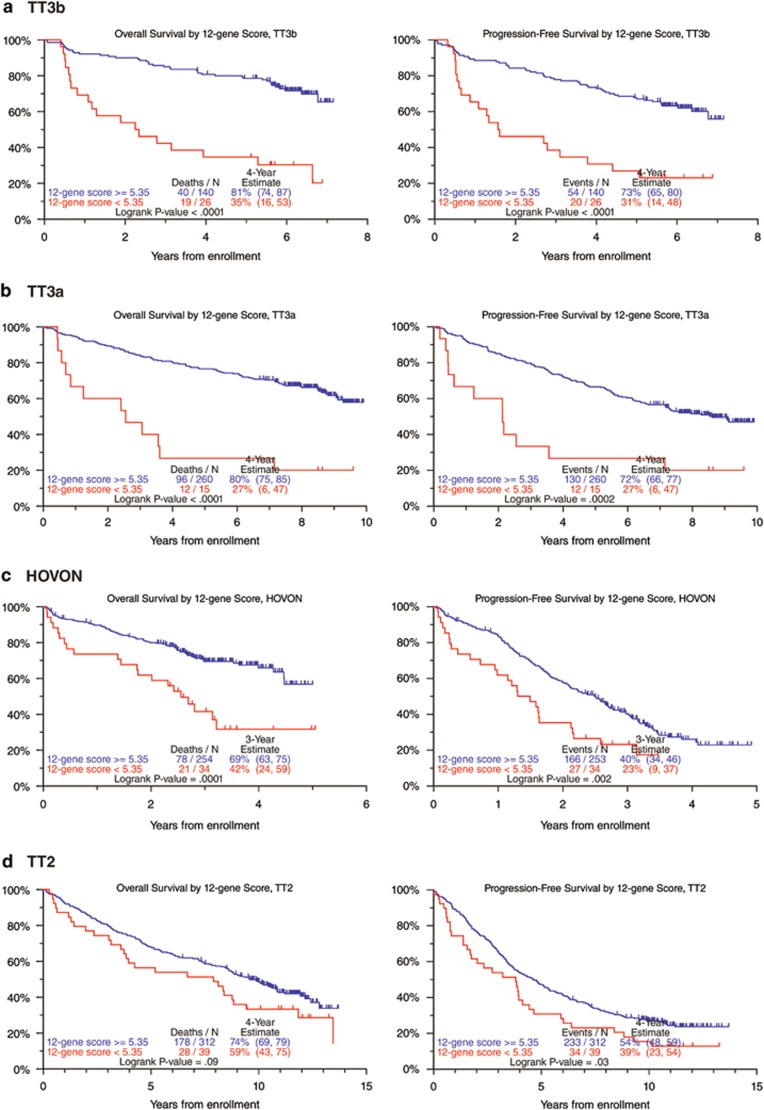
Kaplan–Meier plots of OS and PFS in TT3b (**a**), TT3a (**b**), ComBat-transformed HOVON (**c**) and TT2 (**d**), according to the 12-probeset score for genes associated with CIg.

As low CIg was strongly correlated with a multitude of different prognostic variables and retained independent adversity in the multivariate models 1 and 2 of Table 2, a comparative genomic analysis was carried out to identify gene probes distinguishing low from high CIg cases. Such analysis would enable us to validate our approach in trials where DNA/CIG had not been performed. The Wilcoxon's rank sum test of significance analysis of microarrays of the GEP data for the two groups revealed 12 gene probe sets derived from 11 genes with a *P-*value <10^−4^ and a false discovery rate <10% (Table 4). A risk score (GEP12) was computed from the significant probe sets by subtracting the sum of the expressions of the probes over-expressed in patients with low CIg from the sum of the expressions of the probes under-expressed in patients with low CIg, divided by the total number of probes. Using the running log-rank method, adverse prognostic implications were observed in TT3b for patients exhibiting a GEP12 score <5.35. This GEP12 score <5.35 substituted effectively for low CIg in model 3 of Table 2 and, importantly, dispelled GEP70 high risk and proliferation index. We next examined whether the GEP12 score held prognostic implications in other trials where the doublet discrimination method could not be retrospectively applied or DNA/CIG data were unavailable. Indeed, the GEP12 risk score segregated OS and PFS strongly in the bortezomib-containing TT3b training set (Figure 2a), in test sets of TT3a^6^ (Figure 2b) and in the HOVON65/GMMG-HD4^24^ trials (Figure 2c). In TT2, PFS differed with a strong trend in OS, when both arms were considered combined (Figure 2d).

## Discussion

We show that the presence of low CIg as detected by DNA/CIG is a major adverse prognostic factor in TT3b, even when other GEP-derived prognostic factors were accounted for (Table 2). Although linked to a multitude of standard adverse prognostic factors (Table 3), low CIg survived the multivariate models even in the presence of GEP data. Factors that were not linked to CIg, such as older age and low albumin, retained independent adverse significance. The CI-linked GEP12 score outperformed GEP70-risk in TT3b (see Table 2) and was validated in TT3a, TT2 and HOVON trials. In this trial with contemporary treatment components, DNA/CIG ploidy status (DI) *per se* was not prognostic for either OS or PFS, even when an optimal cutoff point approach for the DI value was obtained (data not shown). We believe that this reflects the improvement in prognosis through newer treatments.^6, 25^

The clinical significance of CIg in MM may be related to its impact on the pathophysiology of the plasma cell. Immunoglobulin-producing and -secreting cells, normal or malignant, are characterized by a low proteasome capacity^26^ that puts the cells under endoplasmic reticulum stress^27^ that is dealt with by the unfolded protein response.^28^ Failure of the plasma cell to mount an effective unfolded protein response in the presence of the immunoglobulin production stress leads to apoptosis.^29, 30^ Bortezomib targets the proteasome and increases endoplasmic reticulum stress.^31^ Consequently, in cases of high CIg signifying high immunoglobulin production, endoplasmic reticulum stress is augmented further by exposure to bortezomib, resulting in accelerated apoptosis regardless of other biologic characteristics of that cell. This hypothesis is supported by the finding that the CIg-derived gene score was significant in the bortezomib-containing TT3a/b and HOVON studies but to a lesser extent in TT2 devoid of a proteasome inhibiting agent. The GEP-defined MS molecular subgroup, corresponding to the t(4; 14) translocation and known to benefit from bortezomib,^6, 32^ was associated with a high CIg in our series (Table 3), thus providing a potential explanation for the sensitivity of this subgroup to proteasome inhibitors.

Low CIg was associated with aggressive disease characteristics (Table 3). Recently, the identification of a subpopulation of MM cells with a reduction in the immunoglobulin production, pre-plasmablastic morphology and immaturity when examined by multicolor flow cytometry has been linked with proteasome inhibition resistance and reduced PFS.^18^ The linkage of low CIg to a high GEP-defined centrosome index is novel. Beyond providing support for the successful completion of the anaphase in eukaryotic cells, centrosomes also serve in the orientation of the cellular cilia^33^ and are hence an integral part of a successful cellular migration,^34^ perhaps facilitating the generation of extramedullary disease.^35^ Interestingly, a centrosome inhibitor has shown promising activity in preclinical models of MM,^36^ thus potentially providing a selective drug for patients with low CIg myeloma.

Of the 12 gene probe sets strongly associated with a low CIg in the Wilcoxon Rank sum test analysis, only 3 were over-expressed (Table 4). Importantly, (204251_s_at) *CEP164*, encoding a centrosomal protein crucial for cilia formation^37^ and not amongst the gene probes forming the centrosome index, had the highest expression in the low CIg group, fitting the association of increased centrosome expression with low CIg (Table 4). Another hyperexpressed gene in the low CIg group was (209776_s_at) SLC19A1, which is one of the GEP70-constituting genes. SLC19A1 is a member of the Solute Carrier (SLC) group of membrane transporters, which encode for a membrane protein that functions as a folate carrier implicated in methotrexate cellular accumulation in pediatric acute lymphoblastic leukemia.^38^ Consequently, under the prism of the recent advances in this class of drugs,^39^ folate antagonists merit a new look in MM with low CIg. The remaining gene with an inverse relation of expression, (227896_at) BCCIP, is involved in cell cycle regulation and it was recently shown that it promotes tumor progression.^40^

Among the 9 gene probes under-expressed in the low CIg group, (213187_x_at and 212788_x_at) *FTL* encodes for the L subunit of the ferritin protein. Recently, the H subunit of the ferritin molecule was linked to predicting sensitivity to bortezomib of myeloma cells *in vitro*.^41^ (215949_x_at) IGHM encodes for the constant part of the heavy mu chain and is a marker of B-cell differentiation, as has also been shown by others.^42^ In a similar fashion, (219117_s_at) FKBP11, a member of the FKBP family of peptidyl-prolyl cis/trans isomerases, has been found to be uniquely highly expressed in MM;^43^ its downregulation in the low CIg group furthers supports the dedifferentiation of the low immunoglobulin-producing plasma cells. (207408_at) SLC22A14, a member of the SLC group of membrane transporters, encodes for a transmembrane small molecule cation transporter,^44^ implying that it could be potentially involved in the intracellular transportation of agents in MM. (217622_at) RHBDD3, otherwise known as PTAG (pituitary tumor apoptosis gene), encodes for a protein that has been shown to be involved in cell cycle regulation and promote apoptosis in solid tumors,^45^ whereas (226286_at) ELMOD encodes for a cytoskeleton protein that recently has been shown to be important in the functionality of stereo-cilia.^46^ Finally, (215432_at) ACSM1 encodes for a protein with a mitochondrial location that is implicated in the metabolism of fatty acids,^47^ and (239844_x_at) C1orf228 encodes for a protein of unknown functionality.^48^

In conclusion, DNA/CIG, a readily applicable, fast and low-cost test, offers valuable prognostic information even in the era of genomic profiling and contemporary therapies. Its incorporation into survival analysis revealed new insights into the disease biology and hitherto unsuspected MM-relevant genes. These genes, when used in a GEP12 risk score, proved to be prognostically powerful in a multitude of MM trials and may provide useful information for the evaluation and establishment of new targeted therapies.

## Figures and Tables

**Table 1:** Patient baseline characteristics

*Factor*	n/N *(%)*
*Clinical parameters*	
Age ⩾65 years	37/139 (27)
Male	86/139 (62)
Caucasian	130/139 (94)
IgG isotype	75/139 (54)
IgA isotype	33/139 (24)
Other	31/139 (22)
Abnormal K/L ratio	133/139 (96)
Involved light chain: K	81/133 (61)
Involved light chain: L	52/133 (39)
Albumin <3.5 g/dl	67/139 (48)
B2M ⩾3.5 mg/l	82/137 (60)
B2M >5.5 mg/l	43/137 (31)
ISS stage 1	35/137 (26)
ISS stage 2	59/137 (43)
ISS stage 3	43/137 (31)
Hb <10 g/dl	43/139 (31)
Creatinine ⩾2 mg/dl	9/139 (6)
CRP ⩾8 mg/l	45/139 (32)
LDH ⩾190 U/l	31/139 (22)
BMPC ⩾33%	93/134 (69)
Cytogenetic abnormalities	57/136 (42)

*GEP parameters*	
GEP70 high risk	32/139 (23)
GEP80 high risk	16/139 (12)
GEP CD-1 subgroup	12/139 (9)
GEP CD-2 subgroup	20/139 (14)
GEP HY subgroup	46/139 (33)
GEP LB subgroup	10/139 (7)
GEP MF subgroup	7/139 (5)
GEP MS subgroup	20/139 (14)
GEP PR subgroup	24/139 (17)
GEP proliferation index ⩾10	16/139 (12)
GEP centrosome index ⩾3	69/139 (50)
GEP 1q21 amplification	64/139 (46)

*DNA/CIG parameters*	
Aneuploid	122/139 (88)
Dominant diploid	53/139 (38)
Dominant hyperdiploid	81/139 (58)
Dominant hypodiploid	5/139 (4)
Number of DNA stem lines=1	25/139 (18)
Number of DNA stem lines=2	97/139 (70)
Number of DNA stem lines >2	17/139 (12)
Any CIg <2.8	60/139 (43)
Total LCR% >50%	28/139 (20)

Abbreviations: B2M, beta-2-microglobulin; CD-1, cyclin D1; CD-2, cyclin D2; CIg, cytoplasmic immunoglobulin index; FDC, DNA and cytoplasmic flow cytometry; GEP, gene expression profile; Hb, hemoglobin; HY, hyperdiploid; ISS, International Staging System; LB, low bone; LCR%, light chain-restricted percentage; LDH, lactate dehydrogenase; MF, MAF/MAFB; PR, proliferation.

*n*/*N* (%) denotes number with factor/number with valid data for factor.

**Table 2:** Cox proportional hazards regression modeling for OS and PFS

*Variable*	n/N *(%)*	*Overall survival*		*Progression-free survival*	
		*HR (95% CI)*	P*-value*	*HR (95% CI)*	P*-value*
*Univariate*					
Age ⩾65 years	37/139 (27)	**2.11 (1.20, 3.72)**	**0.010**	**1.87 (1.12, 3.13)**	**0.017**
Caucasian	130/139 (94)	**0.47 (0.19, 1.20)**	**0.115**	**0.40 (0.18, 0.89)**	**0.025**
IgA Isotype	33/139 (24)	**1.68 (0.93, 3.06)**	**0.087**	**2.10 (1.25, 3.54)**	**0.005**
Involved light chain: K	81/133 (61)	**0.53 (0.30, 0.92)**	**0.024**	**0.49 (0.30, 0.81)**	**0.006**
Albumin <3.5 g/dl	67/139 (48)	**2.85 (1.57, 5.18)**	**<0.001**	**2.11 (1.27, 3.52)**	**0.004**
B2M >5.5 mg/l	43/137 (31)	**1.93 (1.09, 3.40)**	**0.023**	1.65 (0.99, 2.75)	0.056
Hb <10 g/dL	43/139 (31)	**2.07 (1.18, 3.62)**	**0.011**	**1.85 (1.12, 3.06)**	**0.016**
LDH ⩾190 U/l	31/139 (22)	**2.17 (1.20, 3.95)**	**0.011**	**1.89 (1.09, 3.27)**	**0.023**
Cytogenetic abnormalities	57/136 (42)	**2.07 (1.17, 3.65)**	**0.012**	**1.80 (1.09, 2.96)**	**0.021**
GEP70 high risk	32/139 (23)	**4.86 (2.77, 8.53)**	**<0.001**	**3.84 (2.31, 6.38)**	**<0.001**
GEP80 high risk	16/139 (12)	**6.72 (3.62, 12.47)**	**<0.001**	**5.40 (2.99, 9.77)**	**<0.001**
GEP PR subgroup	24/139 (17)	**3.24 (1.80, 5.85)**	**<0.001**	**3.26 (1.91, 5.55)**	**<0.001**
GEP proliferation index ⩾10	16/139 (12)	**4.49 (2.36, 8.52)**	**<0.001**	**4.54 (2.48, 8.30)**	**<0.001**
GEP centrosome index ⩾3	69/139 (50)	**2.75 (1.51, 4.98)**	**<0.001**	**2.33 (1.39, 3.90)**	**0.001**
1q21 Amplification by GEP	64/139 (46)	**1.83 (1.04, 3.22)**	**0.035**	**2.38 (1.43, 3.97)**	**<0.001**
Number of stem lines >2	17/139 (12)	**2.65 (1.35, 5.20)**	**0.005**	**2.01 (1.04, 3.87)**	**0.037**
Any CIg <2.8	60/139 (43)	**2.36 (1.35, 4.15)**	**0.003**	**2.03 (1.24, 3.34)**	**0.005**
Total LCR% >50%	28/139 (20)	**2.43 (1.32, 4.47)**	**0.004**	**1.88 (1.06, 3.32)**	**0.030**
CIg 12-gene score <5.35	24/139 (17)	**4.26 (2.35, 7.71)**	**<0.001**	**3.34 (1.92, 5.82)**	**<0.001**

*Model 1*^a^					
Age ⩾65 years	37/139 (27)	**2.34 (1.32, 4.16)**	**0.004**	**1.96 (1.16, 3.31)**	**0.011**
Albumin <3.5 g/dL	67/139 (48)	**3.02 (1.65, 5.51)**	**<0.001**	**2.21 (1.32, 3.71)**	**0.002**
Any CIg<2.8	60/139 (43)	**2.08 (1.18, 3.67)**	**0.012**	**1.84 (1.11, 3.04)**	**0.017**
R^2^		**0.4023**		**0.2764**	

*Model 2*^b^					
Age ⩾65yr	37/139 (26)	**1.86 (1.04, 3.33)**	**0.036**	1.58 (0.94, 2.68)	0.086
Albumin <3.5 g/dl	68/139 (49)	**2.49 (1.33, 4.65)**	**0.004**	**1.88 (1.11, 3.19)**	**0.019**
IgA Isotype	34/139 (24)	1.35 (0.74, 2.47)	0.328	**1.76 (1.04, 2.99)**	**0.035**
GEP70 high risk	32/139 (23)	**2.51 (1.23, 5.12)**	**0.011**	**2.10 (1.09, 4.07)**	**0.027**
GEP proliferation index ⩾10	16/139 (12)	2.19 (0.99, 4.83)	0.052	**2.40 (1.10, 5.24)**	**0.027**
Any CIg <2.8	60/139 (43)	**2.02 (1.14, 3.58)**	**0.016**	**1.87 (1.13, 3.09)**	**0.015**
R^2^		**0.4761**		**0.3923**	

*Model 3*^c^					
Age ⩾65 years	37/139 (27)	**2.05 (1.14, 3.68)**	**0.017**	**1.80 (1.06, 3.06)**	**0.030**
Albumin <3.5 g/dl	67/139 (48)	**3.78 (2.04, 7.01)**	**<0.001**	**2.68 (1.58, 4.55)**	**<0.001**
CIg 12-gene score <5.35	24/139 (17)	**4.56 (2.44, 8.54)**	**<0.001**	**3.59 (2.00, 6.44)**	**<0.001**
R^2^		**0.4198**		**0.2905**	

Abbreviations: B2M, beta-2 miscroglobulin; CIg, cytoplasmic immunoglobulin index; CI, confidence interval; GEP, gene expression profile; HR, hazard ratio; HY, hyperdiploid; LDH, lactate dehydrogenase; PR, proliferation; LCR%, light chain-restricted percentage.

*P*-value from Wald *χ*^2^-test in Cox Regression. NS2 multivariate results not statistically significant at 0.05 level. All univariate *P*-values reported with a *P*-value ⩽0.1 are shown in bold. Multivariate model uses stepwise selection with entry level 0.1 and variable remains if meets the 0.05 level. A multivariate *P*-value >0.05 indicates variable forced into model with significant variables chosen using stepwise selection.

a Multivariate, CIg included and no GEP variables were allowed for selection.

b Multivariate, all variables allowed for selection and no CI-derived gene probe variable included.

c Multivariate, GEP included and 12-gene score in place of any CIg.

**Table 3:** Logistic regression analysis for factors associated with ‘any CIg <2.8'

*Variable*	N	*Any CIg <2.8*	*Any CIg ≥2.8*	*OR (95% CI)*	P*-value*
*Univariate*					
B2M ⩾3.5 mg/l	137	44/82 (54%)	14/55 (25%)	3.39 (1.61, 7.15)	0.0013
B2M >5.5 mg/l	137	28/43 (65%)	30/94 (32%)	3.98 (1.86, 8.54)	0.0004
Hb <10 g/dl	139	27/43 (63%)	33/96 (34%)	3.22 (1.52, 6.81)	0.0022
CRP ⩾8 mg/l	139	27/45 (60%)	33/94 (35%)	2.77 (1.33, 5.76)	0.0063
LDH ⩾190 U/l	139	21/31 (68%)	39/108 (36%)	3.72 (1.59, 8.69)	0.0025
BMPC% ⩾33%	134	49/93 (53%)	10/41 (24%)	3.45 (1.52, 7.85)	0.0031
Cytogenetic abnormalities	136	30/57 (53%)	28/79 (35%)	2.02 (1.01, 4.05)	0.0468
GEP70 high risk	139	20/32 (63%)	40/107 (37%)	2.79 (1.23, 6.31)	0.0136
GEP80 high risk	139	11/16 (69%)	49/123 (40%)	3.32 (1.09, 10.15)	0.0352
GEP MS subgroup	139	4/20 (20%)	56/119 (47%)	0.28 (0.09, 0.89)	0.0311
GEP centrosome index ⩾3	139	41/69 (59%)	19/70 (27%)	3.93 (1.93, 8.02)	0.0002
Number of stem lines >2	139	11/17 (65%)	49/122 (40%)	2.73 (0.95, 7.87)	0.0628
Total LCR% >50%	139	21/28 (75%)	39/111 (35%)	5.54 (2.16, 14.18)	0.0004

*Multivariate*					
B2M >5.5 mg/l	132	28/42 (67%)	29/90 (32%)	3.04 (1.27, 7.25)	0.0121
CRP ⩾8 mg/l	132	25/42 (60%)	32/90 (36%)	3.35 (1.40, 8.01)	0.0065
GEP centrosome index ⩾3	132	38/65 (58%)	19/67 (28%)	2.56 (1.12, 5.87)	0.0263
LCR% >50	132	20/26 (77%)	37/106 (35%)	4.97 (1.68, 14.72)	0.0038

Abbreviations: B2M, beta-2 microglobulin; BMPC%, bone marrow plasma cell percentage; CRP, C-reactive protein; CI, confidence interval; GEP, gene expression profile; Hb, hemoglobin; LDH, lactate dehydrogenase; MS, MMSET; LCR%, light chain-restricted percentage; OR, odds ratio.

*P*-value from Wald *χ*^2^-test in logistic regression. NS2 multivariate results not statistically significant at 0.05 level. Univariate *P*-values reported if <0.1. Multivariate model uses stepwise selection with entry level 0.1 and variable remains if meets the 0.05 level. A multivariate *P*-value >0.05 indicates variable forced into model with significant variables chosen using stepwise selection.

**Table 4:** List of differentially expressed gene probes with a *q-*value less than 0.1 from the Wilcoxon's rank sum test significance analysis of microarrays of the ‘any CIg <2.8' and ‘all CIg ⩾2.8' groups of patients

*Affymetrix probe*	*Symbol*	*Chromosome*	*Description*	*Mean signal: any CIg <2.8*	*Mean signal: any CIg* ⩾*2.8*	P*-value*	q*-value*
239844_x_at	C1orf228	chr1p34.1	Chromosome 1 open reading frame 228	6.581345	7.389482	1.49E-06	0.019418
213187_x_at	FTL	chr19q13.33	Ferritin, light polypeptide	14.14725	14.52846	1.65E-06	0.019418
215432_at	ACSM1	chr16p12.3	Acyl-CoA synthetase medium-chain family member 1	4.267809	5.102535	1.65E-06	0.019418
226286_at	ELMOD3	chr2p11.2	ELMO/CED-12 domain containing 3	8.116286	8.691678	1.78E-06	0.019418
209776_s_at	SLC19A1	chr21q22.3	Solute carrier family 19 (folate transporter), member 1	6.504695	5.352083	1.11E-05	0.08482
217622_at	RHBDD3	chr22q12.2	Rhomboid domain containing 3	8.329782	8.782621	1.16E-05	0.08482
212788_x_at	FTL	chr19q13.33	Ferritin, light polypeptide	14.7247	15.07511	1.37E-05	0.085441
227896_at	BCCIP	chr10q26.1	BRCA2 and CDKN1A interacting protein	8.344684	7.80806	1.68E-05	0.091845
215949_x_at	IGHM	chr14q32.33	Immunoglobulin heavy constant mu	9.263938	11.03889	2.01E-05	0.09394
219117_s_at	FKBP11	chr12q13.12	FK506 binding protein 11, 19 kDa	14.69882	15.04902	2.30E-05	0.09394
207408_at	SLC22A14	chr3p21.3	Solute carrier family 22, member 14	8.967901	9.309341	2.46E-05	0.09394
204251_s_at	CEP164	chr11q23.3	Centrosomal protein 164kDa	8.27905	7.790321	2.58E-05	0.09394

Abbreviation: CIg, cytoplasmic immunoglobulin index.

With gray background are portrayed the gene probes that are upregulated in the ‘any CIg <2.8' group.

## Notes

BB received research funding from Celgene Corp. and Millennium Pharmaceuticals, Inc. and is a consultant for Celgene Corp., Millennium Pharmaceuticals, Inc., Onyx Pharmaceuticals, Inc., and Amgen, Inc. He is a co-inventor on patents and patent applications related to use of gene expression profiling in cancer medicine that have been licensed to Myeloma Health, LLC, but has no financial interests in this company. The remaining authors declare no conflict of interest.

## References

[bib1] Barlogie B Alexanian R Pershouse M Smallwood L Smith L Cytoplasmic immunoglobulin content in multiple myeloma J Clin Invest 1985 76 765 769 2411762 10.1172/JCI112033 PMC423897

[bib2] Barlogie B Alexanian R Dixon D Smith L Smallwood L Delasalle K Prognostic implications of tumor cell DNA and RNA content in multiple myeloma Blood 1985 66 338 341 2410065

[bib3] Greipp PR Trendle MC Leong T Oken MM Kay NE Van Ness B Is flow cytometric DNA content hypodiploidy prognostic in multiple myeloma Leuk Lymphoma 1999 35 83 89 10512165 10.3109/10428199909145707

[bib4] Garcia-Sanz R Orfao A Gonzalez M Moro MJ Hernandez JM Ortega F Prognostic implications of DNA aneuploidy in 156 untreated multiple myeloma patients. Castelano-Leones (Spain) Cooperative Group for the Study of Monoclonal Gammopathies Br J Haematol 1995 90 106 112 7786771 10.1111/j.1365-2141.1995.tb03387.x

[bib5] Chng WJ Santana-Davila R Van Wier SA Ahmann GJ Jalal SM Bergsagel PL Prognostic factors for hyperdiploid-myeloma: effects of chromosome 13 deletions and IgH translocations Leukemia 2006 20 807 813 16511510 10.1038/sj.leu.2404172

[bib6] Nair B van Rhee F Shaughnessy JD Jr Anaissie E Szymonifka J Hoering A Superior results of Total Therapy 3 (2003-33) in gene expression profiling-defined low-risk multiple myeloma confirmed in subsequent trial 2006-66 with VRD maintenance Blood 2010 115 4168 4173 20124509 10.1182/blood-2009-11-255620 PMC2879104

[bib7] International Myeloma Working G Criteria for the classification of monoclonal gammopathies, multiple myeloma and related disorders: a report of the International Myeloma Working Group Br J Haematol 2003 121 749 757 12780789

[bib8] Barlogie B Anaissie E van Rhee F Pineda-Roman M Zangari M Shaughnessy J The Arkansas approach to therapy of patients with multiple myeloma Best Pract Res Clin Haematol 2007 20 761 781 18070718 10.1016/j.beha.2007.09.005 PMC2234651

[bib9] Zhan F Huang Y Colla S Stewart JP Hanamura I Gupta S The molecular classification of multiple myeloma Blood 2006 108 2020 2028 16728703 10.1182/blood-2005-11-013458 PMC1895543

[bib10] Shaughnessy JD Jr Zhan F Burington BE Huang Y Colla S Hanamura I A validated gene expression model of high-risk multiple myeloma is defined by deregulated expression of genes mapping to chromosome 1 Blood 2007 109 2276 2284 17105813 10.1182/blood-2006-07-038430

[bib11] Shaughnessy JD Jr Qu P Usmani S Heuck CJ Zhang Q Zhou Y Pharmacogenomics of bortezomib test-dosing identifies hyperexpression of proteasome genes, especially PSMD4, as novel high-risk feature in myeloma treated with Total Therapy 3 Blood 2011 118 3512 3524 21628408 10.1182/blood-2010-12-328252 PMC3186329

[bib12] Chng WJ Ahmann GJ Henderson K Santana-Davila R Greipp PR Gertz MA Clinical implication of centrosome amplification in plasma cell neoplasm Blood 2006 107 3669 3675 16373658 10.1182/blood-2005-09-3810 PMC1895774

[bib13] Durie BG Harousseau JL Miguel JS Blade J Barlogie B Anderson K International uniform response criteria for multiple myeloma Leukemia 2006 20 1467 1473 16855634 10.1038/sj.leu.2404284

[bib14] Wersto RP Chrest FJ Leary JF Morris C Stetler-Stevenson MA Gabrielson E Doublet discrimination in DNA cell-cycle analysis Cytometry 2001 46 296 306 11746105 10.1002/cyto.1171

[bib15] Hiddemann W Schumann J Andreef M Barlogie B Herman CJ Leif RC Convention on nomenclature for DNA cytometry. Committee on Nomenclature, Society for Analytical Cytology Cancer Genet Cytogenet 1984 13 181 183 6478442 10.1016/0165-4608(84)90059-1

[bib16] Epstein J Xiao HQ He XY Markers of multiple hematopoietic-cell lineages in multiple myeloma N Engl J Med 1990 322 664 668 2137556 10.1056/NEJM199003083221005

[bib17] Chang CC Schur BC Kampalath B Lindholm P Becker CG Vesole DH A novel multiparametric approach for analysis of cytoplasmic immunoglobulin light chains by flow cytometry Mod Pathol 2001 14 1015 1021 11598172 10.1038/modpathol.3880427

[bib18] Leung-Hagesteijn C Erdmann N Cheung G Keats JJ Stewart AK Reece DE Xbp1s-negative tumor B cells and pre-plasmablasts mediate therapeutic proteasome inhibitor resistance in multiple myeloma Cancer Cell 2013 24 289 304 24029229 10.1016/j.ccr.2013.08.009 PMC4118579

[bib19] Boucher K Parquet N Widen R Shain K Baz R Alsina M Stemness of B-cell progenitors in multiple myeloma bone marrow Clin Cancer Res 2012 18 6155 6168 22988056 10.1158/1078-0432.CCR-12-0531 PMC3500436

[bib20] Crowley J LeBlanc M Jacobson J Salmon SE(eds). Some exploratory tools for survival analysis Proceedings of the First Seattle Symposium in Biostatistics Springer: New York, USA 1997

[bib21] Xu R Oqj A R-squared type measure of dependence for proportional hazards models J Nonparametr Stat 1999 12 83 107

[bib22] Tusher VG Tibshirani R Chu G Significance analysis of microarrays applied to the ionizing radiation response Proc Natl Acad Sci USA 2001 98 5116 5121 11309499 10.1073/pnas.091062498 PMC33173

[bib23] Jeffrey T Leek WEJ Hilary SP Andrew EJ John DS.SVA: Surrogate Variable Analysis. R package version 3.6.0.

[bib24] Sonneveld P Schmidt-Wolf IG van der Holt B El Jarari L Bertsch U Salwender H Bortezomib induction and maintenance treatment in patients with newly diagnosed multiple myeloma: results of the randomized phase III HOVON-65/ GMMG-HD4 trial J Clin Oncol 2012 30 2946 2955 22802322 10.1200/JCO.2011.39.6820

[bib25] Jagannath S Richardson PG Sonneveld P Schuster MW Irwin D Stadtmauer EA Bortezomib appears to overcome the poor prognosis conferred by chromosome 13 deletion in phase 2 and 3 trials Leukemia 2007 21 151 157 17096017 10.1038/sj.leu.2404442

[bib26] Frisan T Levitsky V Masucci MG Variations in proteasome subunit composition and enzymatic activity in B-lymphoma lines and normal B cells Int J Cancer 2000 88 881 888 11093809 10.1002/1097-0215(20001215)88:6<881::aid-ijc7>3.0.co;2-d

[bib27] Pelletier N Casamayor-Palleja M De Luca K Mondiere P Saltel F Jurdic P The endoplasmic reticulum is a key component of the plasma cell death pathway J Immunol 2006 176 1340 1347 16424160 10.4049/jimmunol.176.3.1340

[bib28] Davenport EL Morgan GJ Davies FE Untangling the unfolded protein response Cell Cycle 2008 7 865 869 18414035 10.4161/cc.7.7.5615

[bib29] Auner HW Beham-Schmid C Dillon N Sabbattini P The life span of short-lived plasma cells is partly determined by a block on activation of apoptotic caspases acting in combination with endoplasmic reticulum stress Blood 2010 116 3445 3455 20651073 10.1182/blood-2009-10-250423

[bib30] Cenci S Sitia R Managing and exploiting stress in the antibody factory FEBS Lett 2007 581 3652 3657 17475256 10.1016/j.febslet.2007.04.031

[bib31] Richardson PG Mitsiades C Hideshima T Anderson KC Bortezomib: proteasome inhibition as an effective anticancer therapy Ann Rev Med 2006 57 33 47 16409135 10.1146/annurev.med.57.042905.122625

[bib32] Avet-Loiseau H Leleu X Roussel M Moreau P Guerin-Charbonnel C Caillot D Bortezomib plus dexamethasone induction improves outcome of patients with t(4;14) myeloma but not outcome of patients with del(17p) J Clin Oncol 2010 28 4630 4634 20644101 10.1200/JCO.2010.28.3945

[bib33] Tang N Marshall WF Centrosome positioning in vertebrate development J Cell Sci 2012 125 4951 4961 23277534 10.1242/jcs.038083 PMC3533386

[bib34] Wakida NM Botvinick EL Lin J Berns MW An intact centrosome is required for the maintenance of polarization during directional cell migration PloS One 2010 5 e15462 21203421 10.1371/journal.pone.0015462 PMC3009746

[bib35] Usmani SZ Heuck C Mitchell A Szymonifka J Nair B Hoering A Extramedullary disease portends poor prognosis in multiple myeloma and is over-represented in high-risk disease even in the era of novel agents Haematologica 2012 97 1761 1767 22689675 10.3324/haematol.2012.065698 PMC3487453

[bib36] Raab MS Breitkreutz I Anderhub S Ronnest MH Leber B Larsen TO GF-15, a novel inhibitor of centrosomal clustering, suppresses tumor cell growth *in vitro* and *in vivo* Cancer Res 2012 72 5374 5385 22942257 10.1158/0008-5472.CAN-12-2026

[bib37] Graser S Stierhof YD Lavoie SB Gassner OS Lamla S Le Clech M Cep164, a novel centriole appendage protein required for primary cilium formation J Cell Biol 2007 179 321 330 17954613 10.1083/jcb.200707181 PMC2064767

[bib38] Kager L Cheok M Yang W Zaza G Cheng Q Panetta JC Folate pathway gene expression differs in subtypes of acute lymphoblastic leukemia and influences methotrexate pharmacodynamics J Clin Invest 2005 115 110 117 15630450 10.1172/JCI22477 PMC539195

[bib39] Purcell WT Ettinger DS Novel antifolate drugs Curr Oncol Rep 2003 5 114 125 12583828 10.1007/s11912-003-0098-3

[bib40] Huang YY Dai L Gaines D Droz-Rosario R Lu H Liu J BCCIP suppresses tumor initiation but is required for tumor progression Cancer Res 2013 73 7122 7133 24145349 10.1158/0008-5472.CAN-13-1766 PMC3918420

[bib41] Campanella A Santambrogio P Fontana F Frenquelli M Cenci S Marcatti M Iron increases the susceptibility of multiple myeloma cells to bortezomib Haematologica 2013 98 971 979 23242599 10.3324/haematol.2012.074872 PMC3669455

[bib42] Gutierrez NC Ocio EM de Las Rivas J Maiso P Delgado M Ferminan E Gene expression profiling of B lymphocytes and plasma cells from Waldenstrom's macroglobulinemia: comparison with expression patterns of the same cell counterparts from chronic lymphocytic leukemia, multiple myeloma and normal individuals Leukemia 2007 21 541 549 17252022 10.1038/sj.leu.2404520

[bib43] Frigyesi I Adolfsson J Ali M Kronborg Christophersen M Johnsson E Turesson I Robust isolation of malignant plasma cells in multiple myeloma Blood 2014 123 1336 1340 24385542 10.1182/blood-2013-09-529800

[bib44] Nishiwaki T Daigo Y Tamari M Fujii Y Nakamura Y Molecular cloning, mapping, and characterization of two novel human genes, ORCTL3 and ORCTL4, bearing homology to organic-cation transporters Cytogenet Cell Genet 1998 83 251 255 10072596 10.1159/000015197

[bib45] Bahar A Whitby P Holley S Hoban PR Elder JB Deakin M Primary colorectal tumors fail to express the proapoptotic mediator PTAG and its reexpression augments drug-induced apoptosis Genes Chromosomes Cancer 2007 46 202 212 17117413 10.1002/gcc.20401

[bib46] Johnson KR Longo-Guess CM Gagnon LH Mutations of the mouse ELMO domain containing 1 gene (Elmod1) link small GTPase signaling to actin cytoskeleton dynamics in hair cell stereocilia PloS One 2012 7 e36074 22558334 10.1371/journal.pone.0036074 PMC3338648

[bib47] Iwai N Mannami T Tomoike H Ono K Iwanaga Y An acyl-CoA synthetase gene family in chromosome 16p12 may contribute to multiple risk factors Hypertension 2003 41 1041 1046 12654705 10.1161/01.HYP.0000064944.60569.87

[bib48] Gerhard DS Wagner L Feingold EA Shenmen CM Grouse LH Schuler G The status, quality, and expansion of the NIH full-length cDNA project: the Mammalian Gene Collection (MGC) Genome Res 2004 14 2121 2127 15489334 10.1101/gr.2596504 PMC528928

