# Supplementary material for: The flow cytometry-defined light chain cytoplasmic immunoglobulin index and an associated 12-gene expression signature are independent prognostic factors in multiple myeloma
Source: Leukemia. 2015 Mar 27;29(8):1713–20. doi: 10.1038/leu.2015.65 (PMC4530205; doi:10.1038/leu.2015.65)
Supplement: Supplementary Figures [file leu201565x1.docx]

**Supplementary Figures**

1. **B)**

**C)**
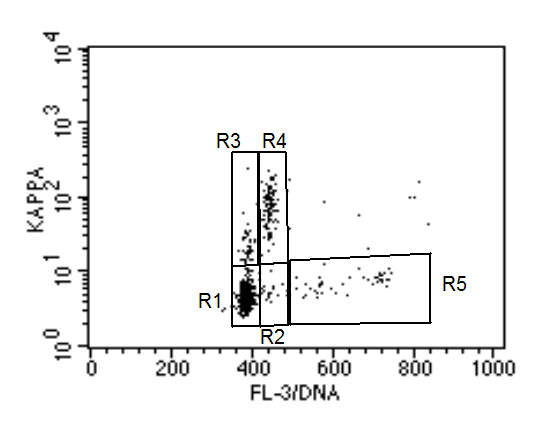

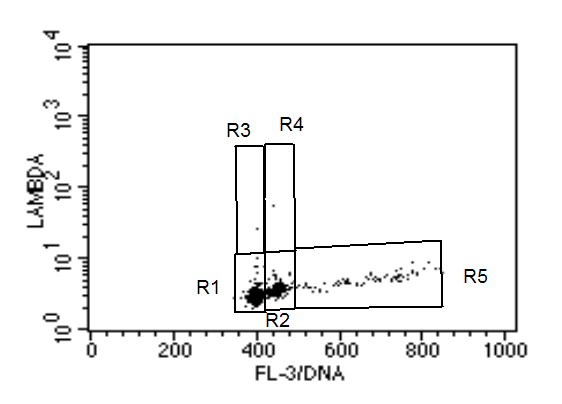


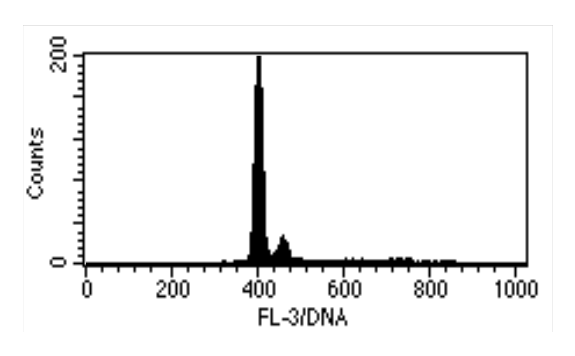


**Figure 1:** DNA/CIG analysis of a kappa light chain positive dominant hyper-diploid stem line. The cytoplasmic light chain fluorescence intensity is depicted on the y-axis and that of the DNA dye on the x-axis. Compare the dot plot for the kappa light chain (Figure 1A) with the dot plot for the lambda light chain (Figure 1B). The DNA Index (DI) was calculated as the ratio of the mean fluorescence channels of light chain-positive versus light chain–negative G_0_/G_1_ cell population. The CIG index (CI) is calculated from the ration of the geometrical means of the cytoplasmic light chain fluorescence intensity on the y-axis (logarithmic scale) of the light chain positive diploid (R3 area) and hyperdiploid (R4 area) to the light chain negative (nonspecific fluorescence) diploid population (R1 area). Two distinct kappa positive stem lines are depicted with high cytoplasmic indexes (CI), a diploid kappa positive population (area R3) with DI=1.0 and CI= 5.2 and a hyper-diploid kappa positive population with DI=1.14 (area R4) and CI= 14.2. Figure 1C depicts the DNA histogram of that population. The red arrow portrays the G_0_/G_1_ hyper-diploid population.

1. **B)**

**C)**

**
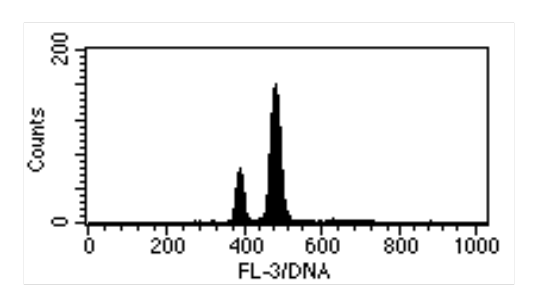
**

**Figure 2:** DNA/CIG analysis of a lambda light chain positive dominant hyper-diploid stem line. The cytoplasmic light chain fluorescence intensity is depicted on the y-axis and that the DNA dye on the x-axis. Compare the dot plot for the kappa light chain (Figure 2A) with the dot plot for the lambda light chain (Figure 2B). The DNA Index (DI) was calculated as the ratio of the mean channels of light chain-positive versus light chain–negative G_0_/G_1_ cell population. The CI is calculated from the ration of the geometrical means of the cytoplasmic light chain fluorescence intensity on the y-axis (logarithmic scale) of the light chain positive diploid (R3 area) and hyperdiploid (R4 area) to the light chain negative (nonspecific fluorescence) diploid population (R1 area) Two distinct lambda positive stem lines are depicted with low cytoplasmic indexes (CI), a diploid lambda positive population (area R3) with DI= 1.0 and CI= 1.8 and a hyper-diploid lambda positive population with DI= 1.24 (area R4) and CI= 2.7. Figure 2C depicts the DNA flow histogram of that population. The red arrow portrays the G_0_/G_1_ hyper-diploid population.

**Figure 3** Kaplan-Meier Plots of Overall Survival **(A)**, Progression Free Survival **(B)**, Cumulative Incidence of Complete Response **(C)** and Complete Response Duration **(D)** for the Total Therapy 3b (TT3b) trial.
