# Supplementary figures and images for: The flow cytometry-defined light chain cytoplasmic immunoglobulin index and an associated 12-gene expression signature are independent prognostic factors in multiple myeloma
Source: Leukemia. 2015 Mar 27;29(8):1713–20. doi: 10.1038/leu.2015.65 (PMC4530205; doi:10.1038/leu.2015.65)

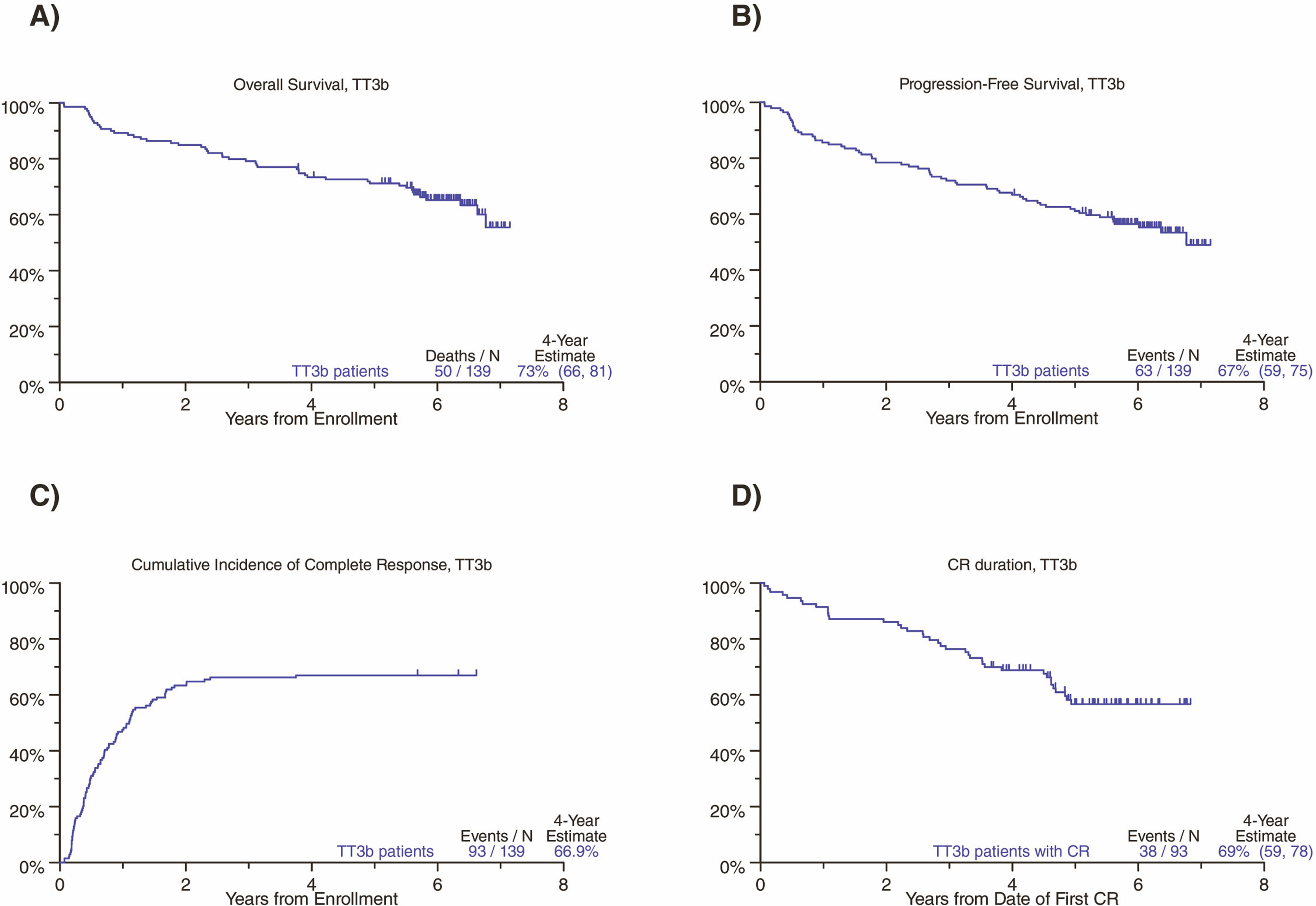

Supplement: Supplementary Figure 3 [file leu201565x2.tif]
